# Easy and accessible way to calibrate a fluorescence microscope and to create a microplastic identification key

**DOI:** 10.1016/j.mex.2023.102053

**Published:** 2023-02-03

**Authors:** Anna Kukkola, Stefan Krause, Yasmin Yonan, Liam Kelleher, Uwe Schneidewind, Gregory H. Sambrook Smith, Holly Nel, Iseult Lynch

**Affiliations:** a School of Geography, Earth and Environmental Sciences, University of Birmingham, Edgbaston, Birmingham B15 2TT, United Kingdom; b Institute for Global Innovation, University of Birmingham, Edgbaston, Birmingham B15 2TT, United Kingdom; c LEHNA- Laboratoire d'ecologie des hydrosystemes naturels et anthropises, University of Lyon, Darwin C & Forel, 3-6 Rue Raphaël Dubois, Villeurbanne 69622, France

**Keywords:** Microplastics, Identification key, Nile Red, Fluorescence microscopy, Particle counting, Fibres, Fragments, Detection, Detection limits, Identification, An easy guide for setting up fluorescence detection limits and a identification key for microplastics identification using Nile Red and fluorescence microscopy.

## Abstract

We present here a technique for setting up detection limits on any fluorescent microscope in conjunction with the fluorophore Nile Red for microplastic identification. Our method also describes a rigorous morphology-specific identification key for microplastics to reduce subjectivity between researchers. The detection limits were established for nine common polymer types and five natural substrates which could result in false-positive signals when using Nile Red for microplastic identification. This method was then applied to real freshwater samples and identified particles were validated with micro-FTIR or Raman spectroscopy. This approach may reduce subjectivity in microplastic identification and counting and enhances transparency, repeatability and harmonization within microplastic research community.

• Instructions for calibration of detection limits for microplastics on fluorescence microscope systems described
• Microplastic identification key developed and tested to reduce false positive detection
• Lower subjectivity for microplastic identification obtained using the detection limits & identification key

## Graphical abstract

**Figure:**
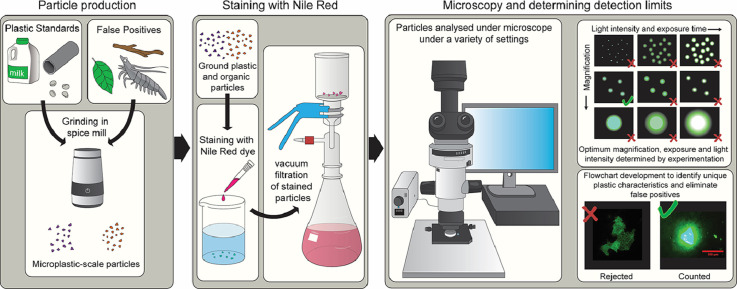

Specifications table

**Table:** 

Subject area	Environmental Science

More specific subject area:	*Microplastic identification for freshwater and sediment samples*
Name of your method:	*An easy guide for setting up fluorescence detection limits and a identification key for microplastics identification using Nile Red and fluorescence microscopy.*
Name and reference of original method:	*Nel, H.A., Chetwynd, A.J., Kelleher,* L.*, Lynch, I., Mansfield, I., Margenat, H., Onoja, S., Goldberg Oppenheimer, P., Sambrook Smith, G.H. and Krause, S. Detection limits are central to improve reporting standards when using Nile Red for microplastic quantification. Chemosphere 263, 127,953 (2021)*https://doi.org/10.1016/j.chemosphere.2020.127953
Resource availability:	*Library of reference images for training purposes*

## Method details

### Background

In microplastic research, a common method for microplastic extraction/fractionation is density separation, frequently carried out using saturated salt solutions [1] followed by visual identification under bright-field microscopy [2,3]. Relying on visual identification is still a very popular technique as analytical approaches such as FT-IR and/or Raman spectroscopy or similar chemical verification methods are not widely accessible and are time consuming and very data intensive [4]. Visual identification, however, is highly subjective, as it depends on individual understanding of how microplastic appears under the light microscope (often based on colour and shape). Visual identification has also been criticized for being imprecise for smaller microplastic particles, especially for sizes below 200 µm [2], [5], [6], [7]. This has led to calls to create reproducible methods that are universally accessible globally. Nile Red staining, a method where samples are stained with fluorescent dye, has been suggested to overcome some of these limitations, as it can be applied at low-cost and is widely accessible [8], [9], [10], [11], [12], [13].

However, this method also has its limitations. Nel et al. (2021) [11] showed that not everything that had fluorescence should be counted as microplastics, as Nile Red can also stain organic matter (OM) present in the samples, which can result in some particles being falsely identified as plastics [11,14]. To overcome this, Nel et al. (2021) [11] investigated the pixel brightness of various plastic polymers and natural substrates before and after Nile Red staining. This allowed a suitable particle brightness (detection limit) to be determined, whereby only particles above this specific threshold would be counted, which would allow detection of microplastics while simultaneously reducing inclusion of false positives.

To set up a fluorescence detection limit, which is optimised for microplastic identification, every microscope used for counting and identifying microplastics must be calibrated. This is because each fluorescence microscope has unique fluorescence intensity/brightness for a specific combination of optical filter(s), light source, detector, and camera [11]. To establish these limits for a specific microscope system, several different settings need to be trialled with known plastic types, as well as some common natural materials (e.g., wood, leaves etc.) that may be present in the samples. This paper provides a guideline for how this calibration process can be carried out, and contributes towards the standardisation of microplastic identification using Nile Red.

Furthermore, to aid reproducibility and transparency we developed easy-to-use identification keys within our research group for use with Nile Red staining. The first key relies on the fluorescence detection limit as a pre-screening for particles of interest to determine which ones should be considered further, thereby considerably shortening the overall time spent on analysing a filter. It then requires the operator to inspect particles above the set fluorescence detection limit under bright-field microscopy and uses morphology specific keys to quantify the different forms present in the sample. The detection limits coupled with the identification keys result in higher detection comparability between individual researchers in terms of microplastic count numbers as well as their morphological categorization. This type of identification/decision tree has been used previously in microplastic literature for bright-field microscopy and is considered an example of best practise (e.g., [15]). Here, we take the next step and combine fluorescence and visual observation to create a quick and robust identification method for microplastic identification and counting.

## Establishing microplastic detection limits

To establish microplastic detection limits in Nile Red supported fluorescent microscopy, thirteen different synthetic polymer types, including polylactic acid (PLA), polyvinyl chloride (PVC), expanded polystyrene (EPS), two types of high-density polyethylene (HDPE), polyamide-6 (PA6), polyethylene (PE), polyethylene terephthalate (PET), three types of polypropylene (PP) and two types of polystyrene (PS), were selected as exemplar materials [16]. Polymer composition was confirmed using thermogravimetric analysis-Fourier transform infrared spectroscopy-gas chromatography-mass spectrometry TGA-FTIR-GCMS [17]. All plastics were ground down with a spice mill and size fractions between 64 µm - 400 µm were kept after sieving with stainless steel sieves, to represent environmental microplastics. Five substrates commonly present in environmental samples were also included as examples of potential false positives. These were wood (twigs), dead leaves (maple and birch), chitosan (chitin) and sand.

Plastic (*n* = 13) and natural substrate (*n* = 5) samples were placed individually into clean glass beakers each with 20 mL of deionised (DI) water and subsequently left to stain with Nile Red at a concentration of 5 µg mL^−1^ for an hour at about 21 °C. Each beaker was gently agitated every 10 min to ensure equivalent staining. Samples were then vacuum-filtered onto Whatmann GF/D glass fibre filters and oven dried for 24 h at 50 °C before imaging. No organic matter digestion was carried out.

**Fig. 1:**
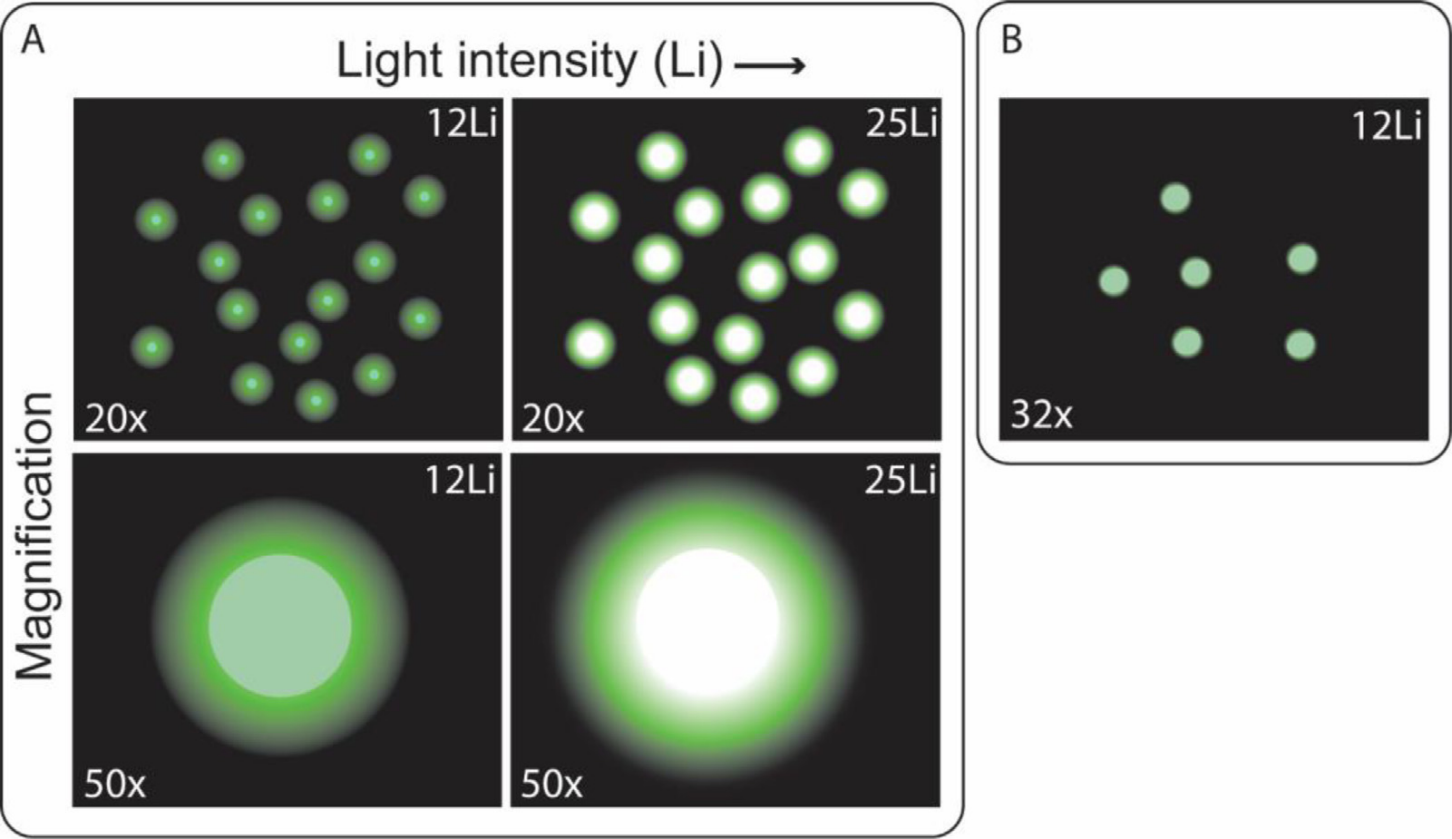
Schematic illustration of general effect of each of the tested system settings on Pixel Brightness (PB). (A) panel shows 20x magnification (top row); Light intensity 12 (2xLi12) and 25 (2xLi25), with 50 ms exposure time and 50x magnification (bottom row); Light intensity 12 (5xLi12) and 25 (5xLi25), with 50 ms exposure time. (B) 32x magnification; Light intensity 12 (3.2xLi12), with 50 ms exposure time.

Imaging was carried out in green fluorescence mode using a macro zoom microscope (Olympus MVX-ZB10) equipped with a 1 × 0.25 N objective (MVPLAPO 1X, Olympus) and a U-M49002XL GFP (Green Fluorescent Protein) filter cube (excitation filter: 470/40 nm, dichroic mirror: 495 nm high pass, emission filter: 525/50 nm) with a 100 W mercury apo lamp (U-HGLGPS, Olympus) as light source. Filters were photographed with a DP74 colour camera. Fifteen particles on each filter were imaged with each of the following five settings (Fig. 1), (i) 20x magnification; Light intensity 12 (2xLi12) and 25 (2xLi25), with 50 ms exposure time, (ii) 50x magnification; Light intensity 12 (5xLi12) and 25 (5xLi25), with 50 ms exposure time, and additionally (iii) 32x magnification; Light intensity 12 (3.2xLi12), with 50 ms exposure time. Fifteen individual particles were imaged per sample type (plastic and false positive type) and each particle was measured for their median green pixel brightness (PB) in arbitrary units (a.u) across their longest Feret diameter using the *CellSens* software under each of the above settings.

Statistical analysis was performed using Rstudio software (Integrated Development for R. Rstudio, PBC, Boston, MA; R Core Team 2021) to evaluate the effects of magnification and light intensity on the PB of the microplastics and natural substrates. Wilcoxon Rank Sum tests with multiple comparisons were performed with PB as the dependant variable and light intensity and magnification as independent variables in order to determine whether the PB of a given sample type changed significantly depending on the settings used.

**Fig. 2:**
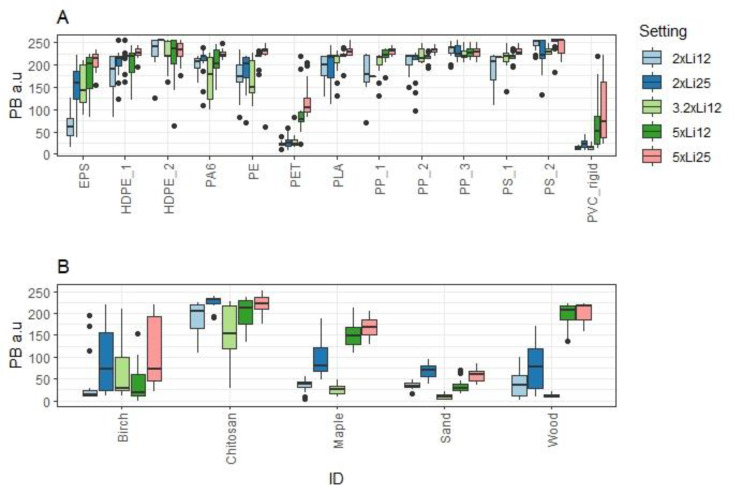
Boxplot illustrating the different pixel brightness (BP) in arbitrary units (a.u) results for (A) thirteen different plastic types and (B) for the five false positive substrates under different magnification, light intensity, exposure time, and image acquisition settings. The black central line in each boxplot indicates the median and whiskers indicate the inter quantile range (25–75%). Settings were as follows: 2xLi12) 20x magnification with light intensity 12 and 50 ms exposure time, 2xLi25) 20x magnification with light intensity 25 and 50 ms exposure time, 3.2xLi12) 32x magnification, light intensity 12, and 50 ms exposure time, 5xLi12) 50x magnification, light intensity 12 and 50 ms exposure time and 5xLi25), 50x magnification, light intensity 25 with 50 ms exposure time.

The results of the Wilcoxon Rank Sum analyses are presented in the supplemental information (SI), but overall, higher magnification and higher light intensity both show an increase in the PB of the detected microplastics, apart from HDPE_2, PP_3 and PS_2 which had high PB regardless of the chosen magnification or light intensity. For PA6, light intensity increased PB significantly, but magnification seemed to have a negligible effect. Conversely, light intensity had no significant impact on the PB of HDPE_1, whereas magnification did. Only for PET microplastic both magnification and light intensity had a significant effect on PB. PET and PVC had the lowest PB, regardless of the magnification used or light intensity settings. Overall, it was impossible to identify a clear statistical trend for the most favourable settings for the microscope in relation to light intensity and magnification. Instead, the descriptive statistics were calculated for each sample type and used to identify the lowest PB cut-off limit by comparing the mean PB value and the lower limit of the 95% confidence interval for each of the five settings, to identify a PB limit as the threshold above which over 90% of all the measured microplastic particles would be identified. The results are shown in a boxplot (Fig. 2A).

The most promising settings for imaging of the microplastics were the higher magnification (50x) and light intensity 12 or 25. With these settings, 90% of all microplastic particles (including PET and PVC) were detectable at a PB limit of 130 a.u. However, imaging of the five natural substrate sample types revealed that choosing this higher magnification (x50) leads to a higher inclusion of false positives, including wood litter (Fig. 2B), which may be abundant in environmental samples, suggesting that lower (32x magnification) should be used.

Another setting that performed well was 32x magnification under light intensity 12 with 50 ms exposure time (Fig. 2A and B, Figures S1 & S2). This setting allowed us to identify 97.5% of plastics (excluding PET and PVC) while only 22.3% of false positives were detectable above the threshold of 100 a.u (Fig. 2). From the detected false positives all were either chitosan or birch, substrate types that are easy to identify as false positives if the identification key described below is used, due to their internal structures. This led to adoption of 100 a.u as our fluorescence intensity threshold, whereby particles and fibres exhibiting intensity higher than this should be considered further for microplastic identification with the identification keys described below.

**Fig. 3:**
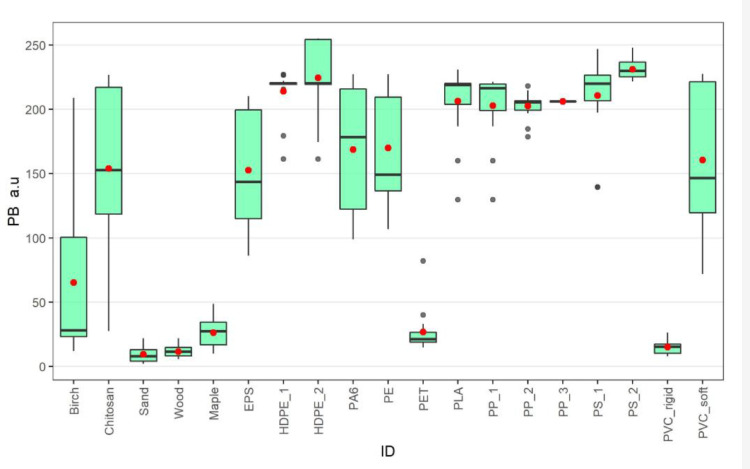
Pixel brightness (PB) in arbitrary units (a.u) for tested samples under 32x magnification, light intensity of 12 and exposure time of 50 ms. The black line indicates the median, and red dots represent mean values for measured PB, while black dots show outliers.

The rigid PVC sample tested seemed to stain relatively poorly with Nile Red. To better understand this, we tested another type of PVC. Plasticised, soft PVC tubing was cut into smaller pieces, ground, and the steps above were repeated with the chosen settings (32x magnification, light intensity 12 and 50 ms exposure). Our results show that soft PVC was easily detectible with fluorescent microscopy, as the mean PB value was 160 a.u. and 87% of all particles were found to be fluorescent above the set 100 a.u. limit (Fig. 3). We hypothesise that the poor and uneven staining of the rigid PVC was due to its high crystalline melting temperature and/or colourants used in products containing this plastic type [11,18]. It is recommended that further research should focus on improving our understanding of how Nile Red reacts with different polymer surfaces.

## Microplastic identification key

To ensure that clear steps were taken to improve reproducibility and to increase transparency, an identification key was created to be used in conjunction with the fluorescence detection limit of 100 a.u set above by the researchers in our facility. It is presented here and in the supplementary information (SI), as an example for the research community with the aim to enhance reproducibility and repeatability in the future.

**Fig. 4:**
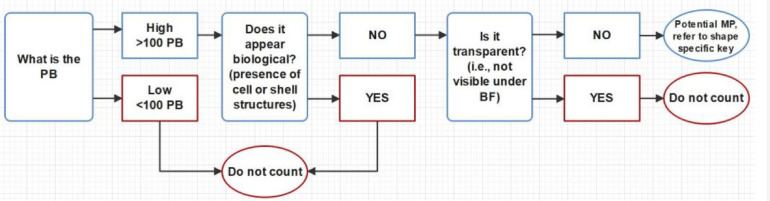
General part of the microplastics identification key that all microplastics had to adhere to. Note: ‘is it transparent’ refers in this context to a highly fluorescent particle without any visible biological structure, which becomes essentially invisible for the researcher under bright-field, making positive identification impossible (PB – pixel brightness, BF – bright-field microscopy, MP - microplastic).

The key comprises four parts, a general part that describes an overall decision on whether a certain particle should be considered further (Fig. 4), and three morphology-specific identification keys to be applied in bright-field mode, which can be found in the SI: fibres (Fig. S3), spheres/beads (Fig. S4) and fragments, which includes films (Fig. S5). Building this extra layer into the identification key to account for morphology adds to the quantification accuracy. Some examples of particles identified in natural samples using the identification key are also included in the images presented in Figure S6 in order to illustrate some of the descriptions provided.

## Method application to freshwater samples

To evaluate the ease with which the identification key and fluorescence detection limits can be applied to quantification of microplastics extracted from freshwater samples by different researchers, two independent researchers working on two separate environmental sample sets were asked to apply the fluorescence detection limits set here, as well as the microplastic identification key detailed above and in the SI. Both researchers underwent training for a day on the microscope, after which they proceeded with counting and identifying putative microplastics in riverbed sediment and water samples according to the method set above. The sequence of the method was: (1) screen the filter under fluorescence mode for particles with light intensity above 100 a.u.; (2) follow the identification keys for any particle above 100 a.u to rule out false positives and confidently identify any microplastics; and (3) screen the filter for fibres only under bright-field mode and use the fibre key for assessment of any fibres detected.

The first researcher had five riverbed sediment samples and the second researcher had five river surface water samples. A minimum of 25% of all the particles identified according to this method were then further analysed for their chemical make-up.

The first researcher was asked to pick roughly 30% of putative microplastic fibres and fragments from the five filters that were sampled and extracted from riverbed sediment. In total, this amounted to 18 out of 78 identified potential microplastics. These were then filtered onto 25 mm silver membranes and analysed with a Bruker Hyperion 2000 microscope, coupled with a Vertex 70 FT-IR spectrometer at the University of East Anglia (UK). The 20 × Ge-tipped ATR lens was used for spectral acquisition and identities were assigned by spectral searching in a Bruker polymers database (Details see [8]). Hit quality Index was set to minimum 60%, but peak position was prioritised over peak intensity. In total the researcher picked nine fibres and nine fragments. One particle and one fibre got lost during transfer onto the silver filter(s), leaving eight fibres and eight fragments available for analysis. The identification results are shown in Table 1 below.

**Table 1:** Results from FTIR analysis for the microplastic particles chosen by Researcher One for chemical analysis for identification of polymer-types.

		PE	PP	PA	PET	lost	Weak spectrum	Cellulose (Possible synthetic)	sum	Plastic ID
Fibre (*n* = 9)		**1**	**0**	**4**	**1**	**1**	**1**	**1**	**9**	**6**
Fragment (*n* = 9)		**4**	**4**	**0**	**0**	**1**	**0**	**0**	**9**	**8**

Researcher Two was similarly asked to pick roughly 30% of particles that they had identified as microplastics according to this method (in total 25 particles out of 98 particles). These were transferred onto anodiscs (Whatman, 25 mm diameter, 0.2 µm pore size) for Raman spectroscopy. A Renishaw InVia Qontor Raman microscope equipped with a 785 nm laser was used for chemical verification. The objective used was 10x with slit width 65 µm, 1200 l/mm, spatial resolution < 1 µm and a spectral resolution < 1 cm^−1^, laser intensity 10% of the system (approximately 15 mW). 10 accumulations of 1 s exposures were repeated three times per particle. Spectral pre-processing for baseline and signal smoothing were implemented in Spectragryph v1.2.15.1 [19], using the advanced baseline function (coarseness = 10 and offset = 0) and the advanced smoothing function (Savitsky-Golay filter with window = 10 and order = 3). The processed spectra were then matched using the Pearson correlation coefficient for the fingerprint region to the reference libraries SLOPP, SLOPP-E [19] and in-house built reference libraries. The results are shown in Table 2.

**Table 2:** Results from Raman spectroscopy for microplastic particles chosen by Researcher Two for confirmatory chemical analysis of polymer type.

Morphology & Number picked	PP	PET	PVC	PEST	PE	Unclassified/burnt	sum	Plastic ID
Fibre (*n* = 12)	**0**	**7**	**0**	**1**	**0**	**3**	**11**	**8**
Fragment (*n* = 13)	**4**	**4**	**5**	**0**	**0**	**3**	**16**	**13**

Of the nine putative microplastic fragments picked by the first researcher, eight were identified as microplastics (four PE, four PP) while one particle was lost during processing. From the nine fibres, again one particle was lost, while six were identified as plastics (four PA, one PE, one PET), one as cellulose and one fibre was unclassified. For the putative microplastics picked by the second researcher, from thirteen selected fragments, all thirteen were identified as plastic (four PE, four PET, five PA) with three more particles showing up in the Raman analysis as unclassified while for the twelve selected fibres, eight were identified as plastic (seven PET, one polyester (PEST)), one particle was lost and three were unclassified. For our examples, we see that based on the fluorescence limit and the identification key, between 70 and 100% of the selected particles were validated to be microplastics with FTIR or Raman spectroscopy. This suggests that our method combining well-established fluorescence limits with a rigorous identification key is generally reliable. Any excess particles picked up during Raman spectroscopy are, in our example, probably organic material transferred to the anodiscs along with the microplastics. However, full-filter scanning with Raman spectroscopy on the original GF/D filters would have taken excessively long and future research should focus on how Raman spectroscopy can be used in fluorescence mode, to aid in the selection of particles for chemical analysis. Researcher Two was also able to select PVC and PEST (not originally tested for) following the above method, leading us to the conclusion that our method although simple is rather robust.

## Additional information

These results clearly highlight that a fluorescence detection limit of 100 a.u was efficient in identifying up to 90% of plastic particles. However, even when the detection limits are set some false positives do still occur, and it has been reported that Nile Red does not stain some synthetic fibres well [20,21]. This implies that while Nile Red is valuable for choosing particles for further screening, it needs to be coupled with methodological visual identification/verification in order to eliminate naturally occurring particles. This can take the form of an identification key with different types of visual cues to determine if something should be counted as microplastic or not. For example, as suggested by [20], some natural fibres, such as cotton and linen, may stain with Nile Red. However, the occurrence of these natural fibres as false positives can be easily countered when fibres are inspected further: natural fibres typically exhibit distinct morphology, such as being internally segmented or twisted around themselves (ribbon-like) [22,23]. It is also suggested herein that in order to counter the potential for false negatives, such as synthetic fibres whose presence may be missed due to poor staining, as a last step the filter will be scanned under bright-field following the identification key established for fibres. Decision/identification keys can facilitate transparency in the microplastic research community and add to the reproducibility of results. The identification key that was created based on relevant literature [15], [23], [24], [25], [26] and the expertise and day-to-day experience of University of Birmingham researchers is demonstrated in the SI as an example.

**Fig. 5:**
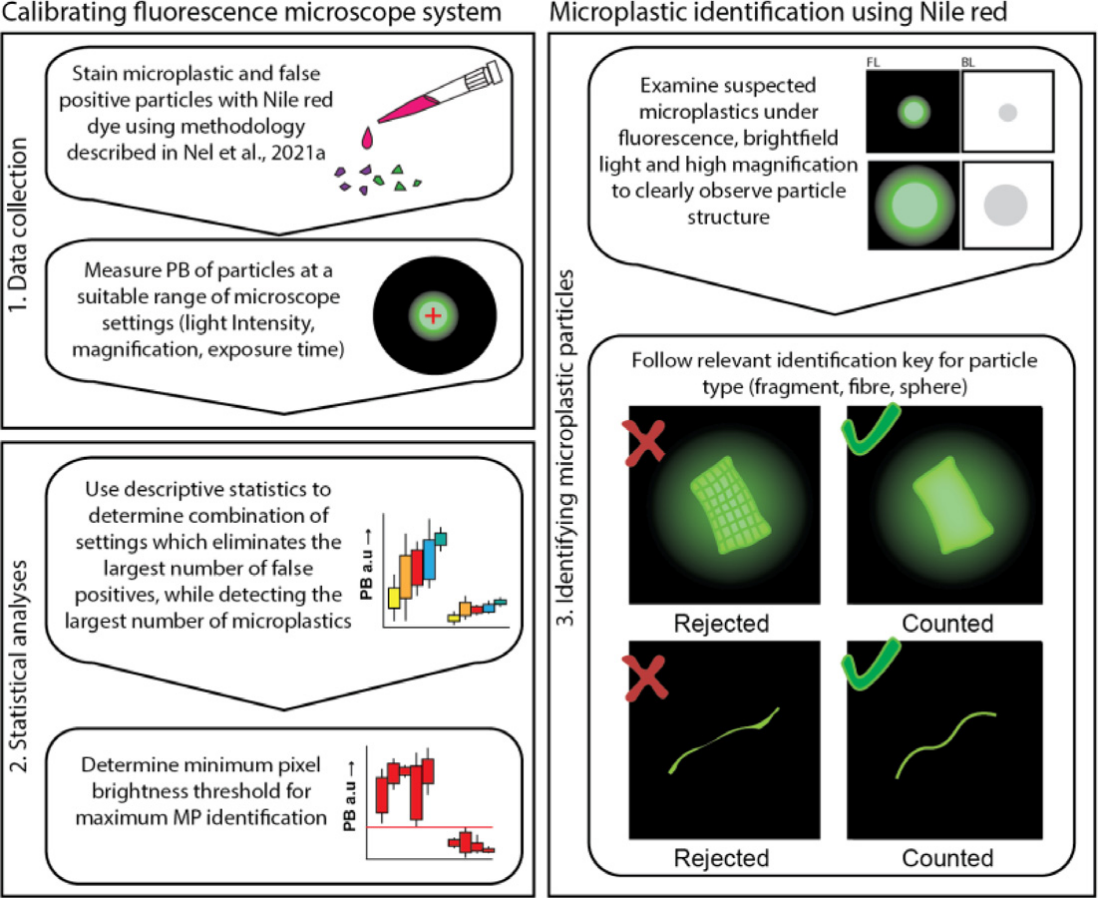
A schematic framework for the workflow described in this paper. (1) Data collection for the calibration of the fluorescence microscope, (2) Use of statistical analysis to identify a suitable threshold whereby most of the microplastics will be detected and most of false positives will not. (3) Use the determined pixel brightness (PB) threshold for pre-screening of particles of interest and develop/adjust existing microplastic identification key(s) to reduce false positive detection, e.g., of biological entities.

The results presented here indicate that the pre-set microplastic selection criteria, which rely not only on the fluorescence detection limit, but also consider morphological cues via the identification key, provide a reliable, fast, and transparent tool for microplastic identification. We propose this approach to be implemented for the microplastic community, as it can lead to more reproducible and consistent results. It should be noted that each new fluorescence system/microscope requires individual calibration to determine its optimal detection limits, as described above. Re-analysis of older samples might also be beneficial, as in many cases there has been over-identification of fibres at the expense of fragments and other shapes, which the identification-tree presented here also helps to overcome. The overall workflow for setting fluorescence detection limits and how to use identification key is presented in Fig. 5.

## CRediT authorship contribution statement

**Anna Kukkola:** Conceptualization, Methodology, Validation, Formal analysis, Investigation, Visualization, Writing – original draft, Writing – review & editing. **Stefan Krause:** Conceptualization, Funding acquisition, Writing – review & editing, Supervision. **Yasmin Yonan:** Methodology, Investigation, Visualization, Writing – review & editing. **Liam Kelleher:** Investigation, Writing – review & editing. **Uwe Schneidewind:** Writing – review & editing. **Gregory H. Sambrook Smith:** Funding acquisition, Supervision. **Holly Nel:** Methodology, Writing – review & editing. **Iseult Lynch:** Conceptualization, Funding acquisition, Writing – review & editing, Supervision.

## Declaration of Competing Interest

The authors declare that they have no known competing financial interests or personal relationships that could have appeared to influence the work reported in this paper.

## Data Availability

Data will be made available on request.

## Appendix Supplementary materials

Image, application 1
