# Supplementary material for: Easy and accessible way to calibrate a fluorescence microscope and to create a microplastic identification key
Source: MethodsX. 2023 Feb 3;10:102053. doi: 10.1016/j.mex.2023.102053 (PMC9941126; doi:10.1016/j.mex.2023.102053)
Supplement: Supplementary file 1 [file mmc1.docx]

**Supplementary data**

The raw results of the Wilcoxon Rank Sum tests with multiple comparisons for different system settings for each different plastic types are provided. The intersection of vertical and horizontal settings shows the pairwise comparison between the setting types and the corresponding p-value. The codes on the rows & columns correspond to the settings used for imaging: **2xLi12)** 20x magnification with light intensity 12 and 50 ms exposure time, **2xLi25)** 20x magnification with light intensity 25 and 50 ms exposure time, **3.2xLi12)** 32x magnification, light intensity 12, and 50 ms exposure time, **5xLi12)** 50x magnification, light intensity 12 and 50 ms exposure time and **5xLi25)**, 50x magnification, light intensity 25 with 50 ms exposure time.

Expanded polystyrene, (EPS)

2xLi12 2xLi25 3.2xli12 5xLi12

2xLi25 5.1e-05 - - -

3.2xli12 5.8e-06 1.00000 - -

5xLi12 4.3e-06 0.11368 0.11368 -

5xLi25 1.3e-07 0.00028 0.00037 0.11368

High-density polyethylene, HDPE

2xLi12 2xLi25 3.2xli12 5xLi12

2xLi25 0.0875 - - -

3.2xli12 0.0031 0.2234 - -

5xLi12 0.0875 0.7437 0.4641 -

5xLi25 8.8e-05 0.0142 0.0780 0.0875

High-density polyethylene from a milk bottle, HDPE_milk

2xLi12 2xLi25 3.2xli12 5xLi12

2xLi25 0.63 - - -

3.2xli12 0.57 0.57 - -

5xLi12 0.65 0.57 0.97 -

5xLi25 0.65 0.57 0.65 0.97

Polyamide-6 fragment, PA-6_frag

2xLi12 2xLi25 3.2xli12 5xLi12

2xLi25 0.012 - - -

3.2xli12 0.769 0.769 - -

5xLi12 0.769 0.769 0.875 -

5xLi25 6.5e-05 0.058 0.769 0.582

Polyethylene red colour, PE_red

2xLi12 2xLi25 3.2xli12 5xLi12

2xLi25 0.27107 - - -

3.2xli12 0.90246 0.48477 - -

5xLi12 0.00065 0.00328 0.00234 -

5xLi25 0.00021 0.00021 0.00021 0.00195

Polyethylene terephthalate, PET

2xLi12 2xLi25 3.2xli12 5xLi12

2xLi25 0.9296 - - -

3.2xli12 1.0000 1.0000 - -

5xLi12 2.9e-06 8.3e-06 5.6e-05 -

5xLi25 6.4e-08 6.4e-08 8.3e-06 0.0064

Polylactid acid, PLA

2xLi12 2xLi25 3.2xli12 5xLi12

2xLi25 0.32297 - - -

3.2xli12 0.06897 0.78509 - -

5xLi12 0.00030 0.06089 0.03077 -

5xLi25 1.5e-06 0.00034 0.00074 0.02847

Polypropylene blue coloured, PP_blue

2xLi12 2xLi25 3.2xli12 5xLi12

2xLi25 1.00000 - - -

3.2xli12 0.63369 0.37149 - -

5xLi12 0.01816 0.35714 0.03847 -

5xLi25 0.00068 0.20833 0.00068 0.11106

Polypropylene grey coloured, PP_grey

2xLi12 2xLi25 3.2xli12 5xLi12

2xLi25 0.0973 - - -

3.2xli12 0.0925 0.0214 - -

5xLi12 0.1126 1.0000 0.0014 -

5xLi25 1.3e-07 4.2e-05 1.5e-07 7.0e-05

Polypropylene white coloured, PP_white

2xLi12 2xLi25 3.2xli12 5xLi12

2xLi25 0.79 - - -

3.2xli12 0.62 0.53 - -

5xLi12 0.53 0.87 0.53 -

5xLi25 0.53 0.86 0.53 0.97

Expanded polystyrene, PS_exp

2xLi12 2xLi25 3.2xli12 5xLi12

2xLi25 0.80000 - - -

3.2xli12 0.07348 0.8000 - -

5xLi12 0.03752 0.8000 0.8000 -

5xLi25 1.3e-07 0.20833 0.04091 0.00036

Polystyrene orange coloured, PS_orange

2xLi12 2xLi25 3.2xli12 5xLi12

2xLi25 0.129 - - -

3.2xli12 0.853 0.910 - -

5xLi12 0.514 0.098 0.514 -

5xLi25 0.862 0.129 0.910 0.910

Polyvinyl chloride rigid, PVC_rigid

2xLi12 2xLi25 3.2xli12 5xLi12

2xLi25 0.014 - - -

3.2xli12 0.713 0.033 - -

5xLi12 3.9e-05 0.024 8.6e-05 -

5xLi25 1.3e-07 8.8e-05 2.6e-07 0.129


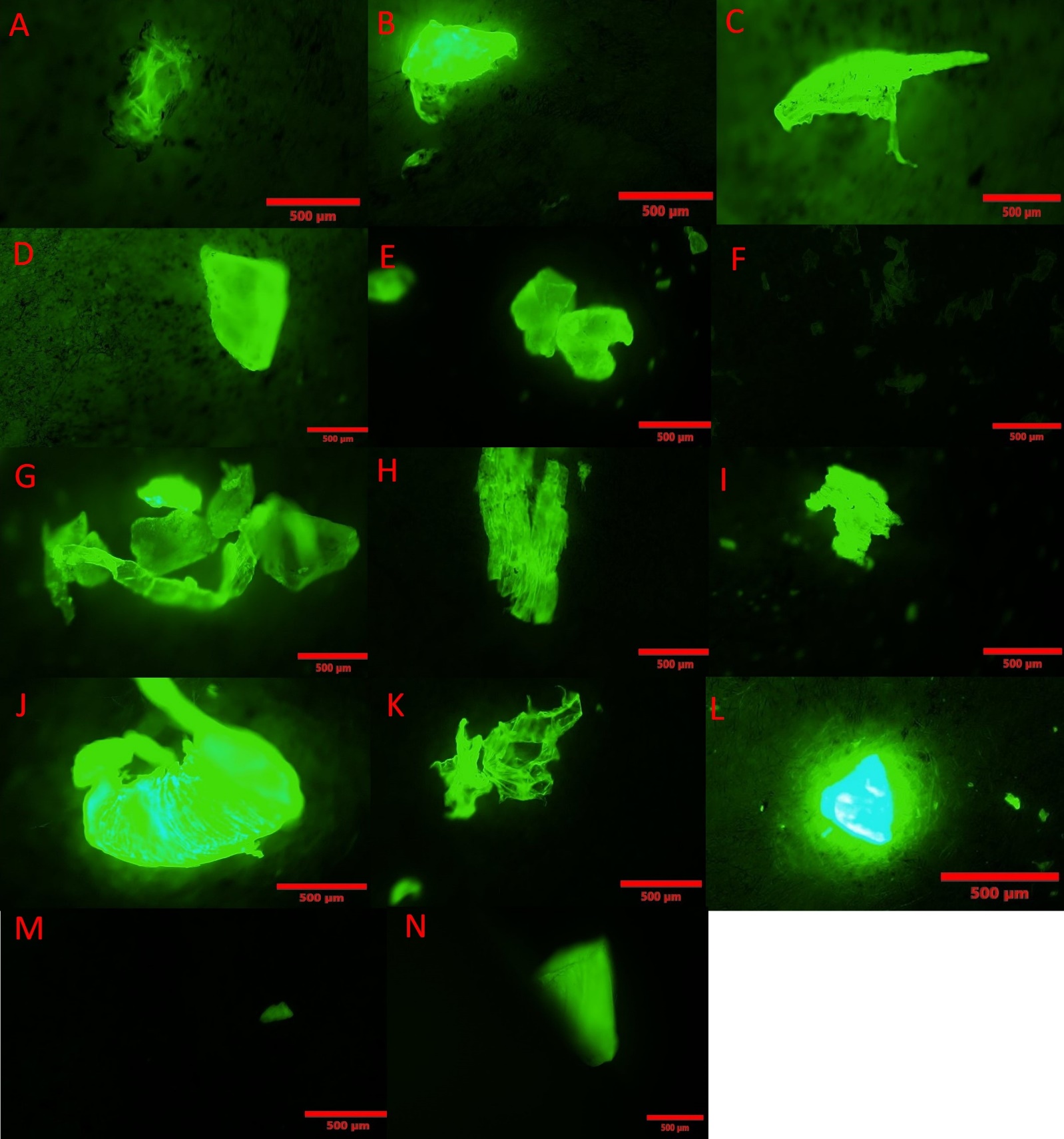


Figure S1: Plastic standards photographed under 32x magnification, Light intensity 12 and with 50 ms exposure time. Plastics are: A) expanded polystyrene (EPS), B) high density polyethylene (HDPE), C) high density polyethylene (HDPE) from a milk bottle, D) polyamide-6 (PA-6), E) polyethylene (PE), F) polyethylene terephthalate (PET), G) polylactic acid (PLA), H) blue polypropylene (PP), I) grey polypropylene (PP), J) white polypropylene (PP), K) polystyrene (PS), L) orange polystyrene (PS), M) rigid polyvinyl chloride (PVC), N) soft polyvinyl chloride (PVC).


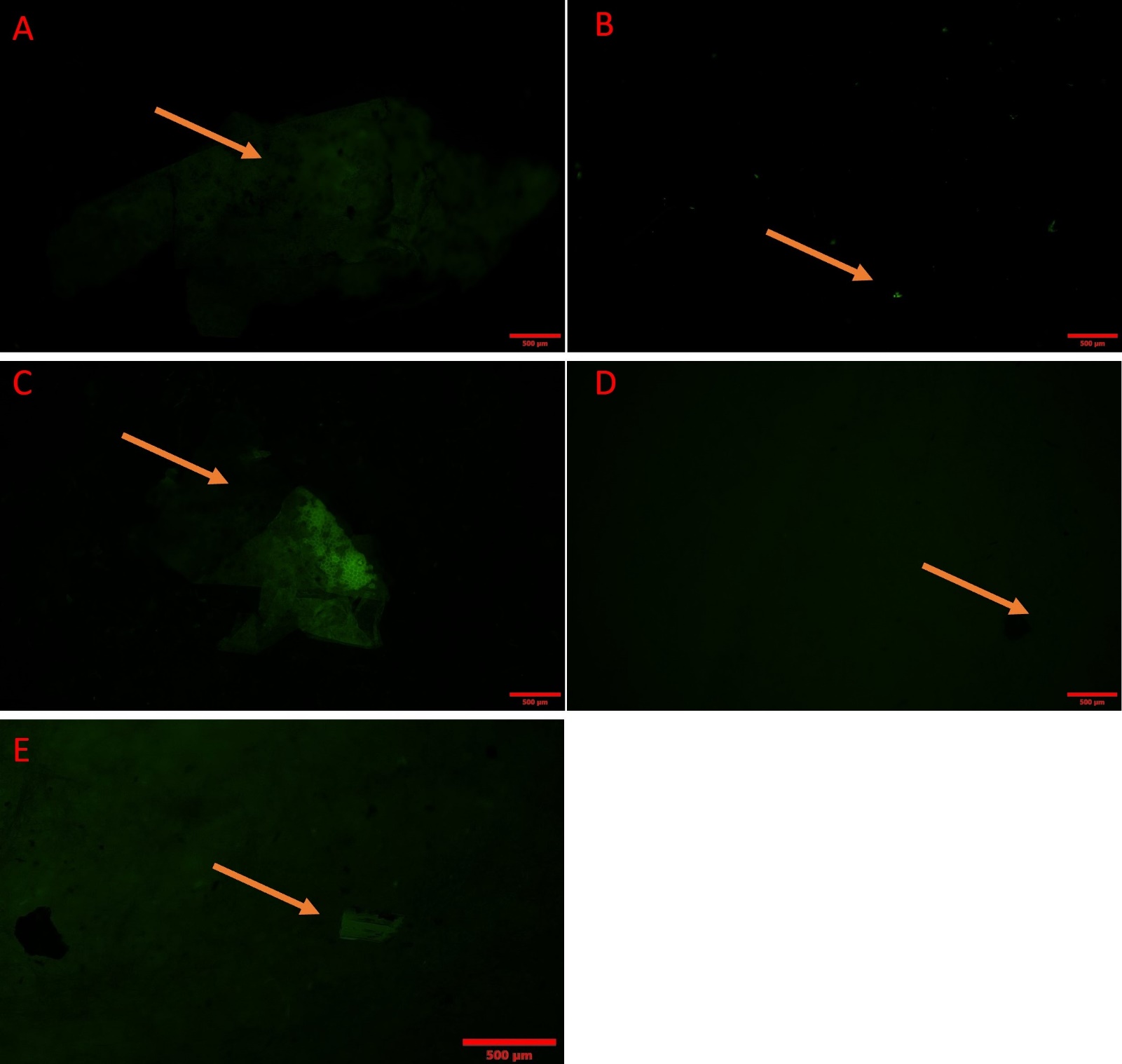


Figure S2: False positives photographed under 32x magnification, light intensity 12 and 50 ms exposure time. A) Birch, B) Chitosan, C) Maple, D) Sand, E) Wood. Orange arrows indicate the locations of the particles of interest.


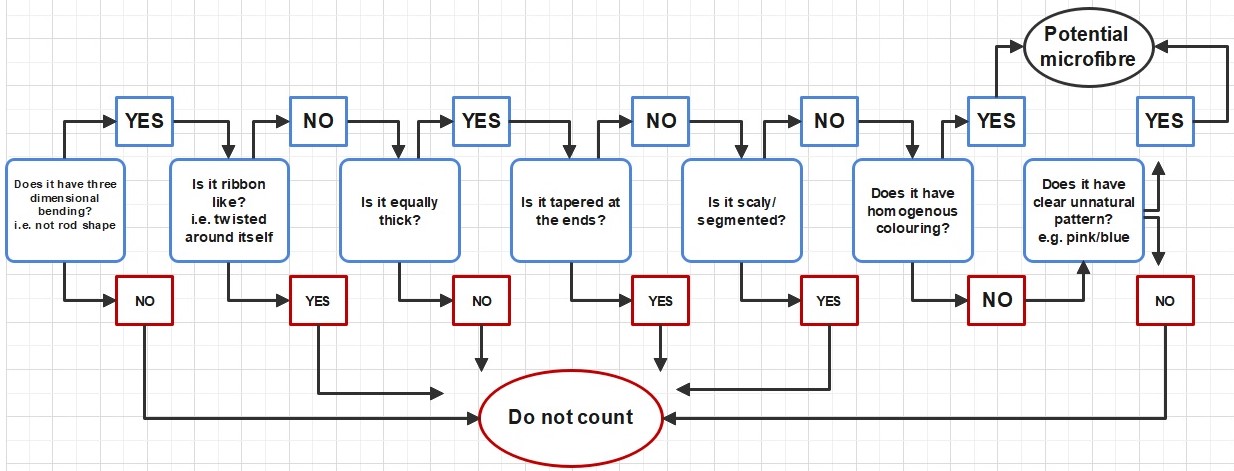


Figure S3: Identification key for fluorescent and non-fluorescent microplastic fibres. It is suggested that each filter should be also scanned under bright field mode for non-fluorescent fibres, as they stain poorly and may lead to false negatives if not included in the final count.


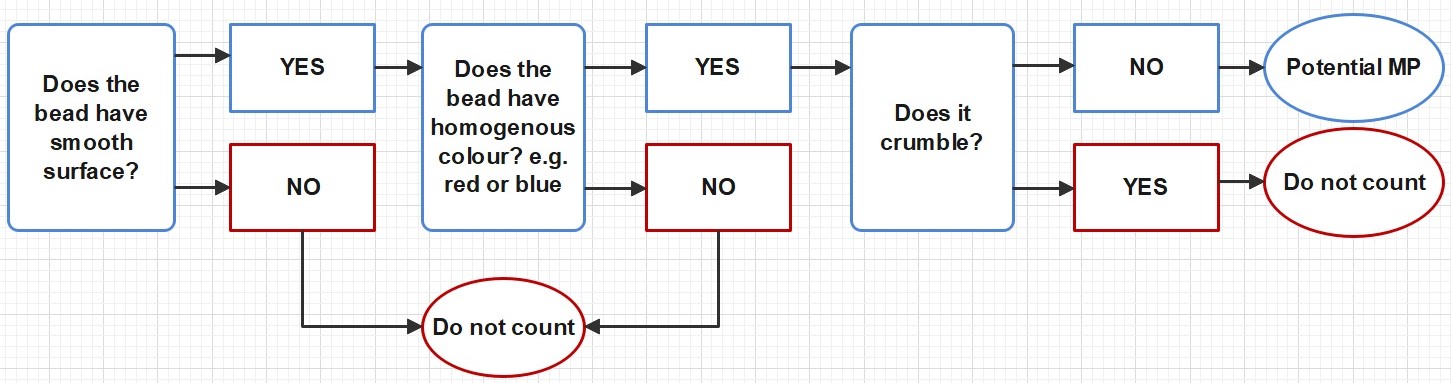


Figure S4: Identification key for microplastic spheres/beads. In our studies we found that eggs which do not have a clear cell structure (typically excluded with the general identification key shown in Figure 5) can exhibit bright fluorescence. However, when poked with a needle/fine forceps they crumble or are filled with fluid, making identification easy.

**
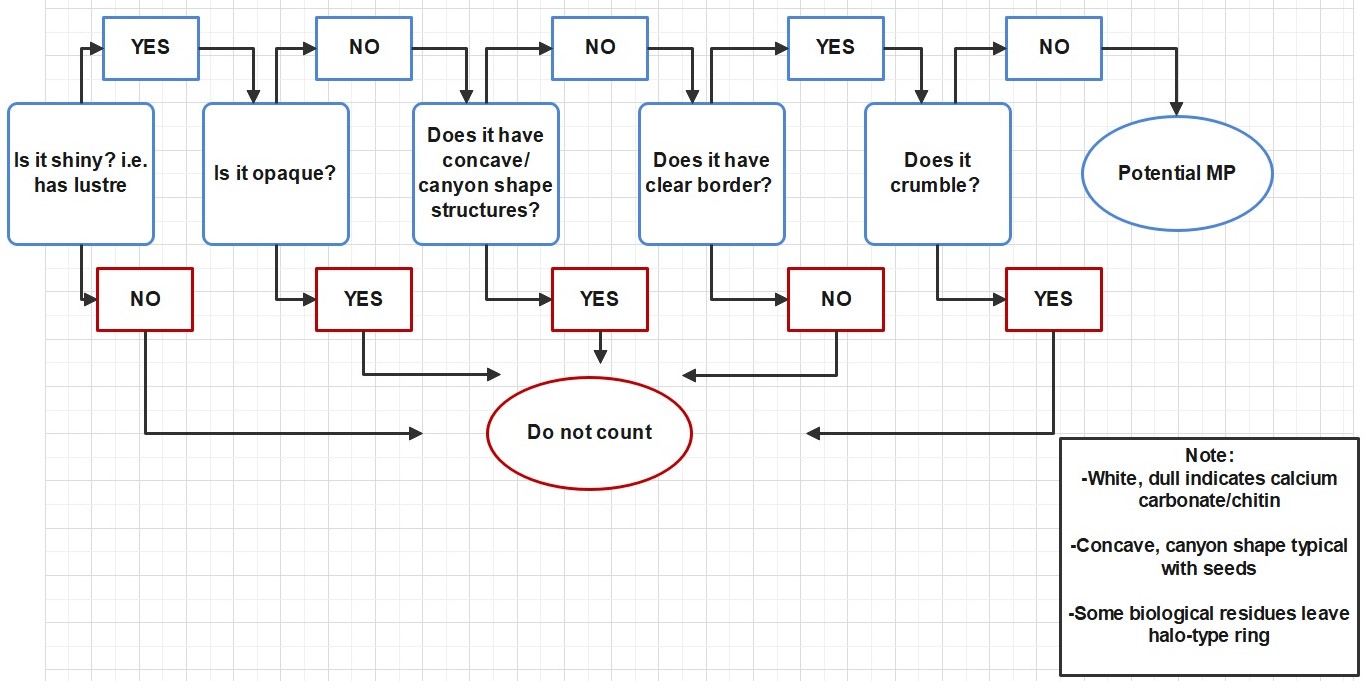
**

Figure S5: Identification key for microplastic fragments including films. Many seeds can exhibit bright fluorescence, with no clear cell structure, but they can be identified by their concave structure, or they may resemble a canyon.


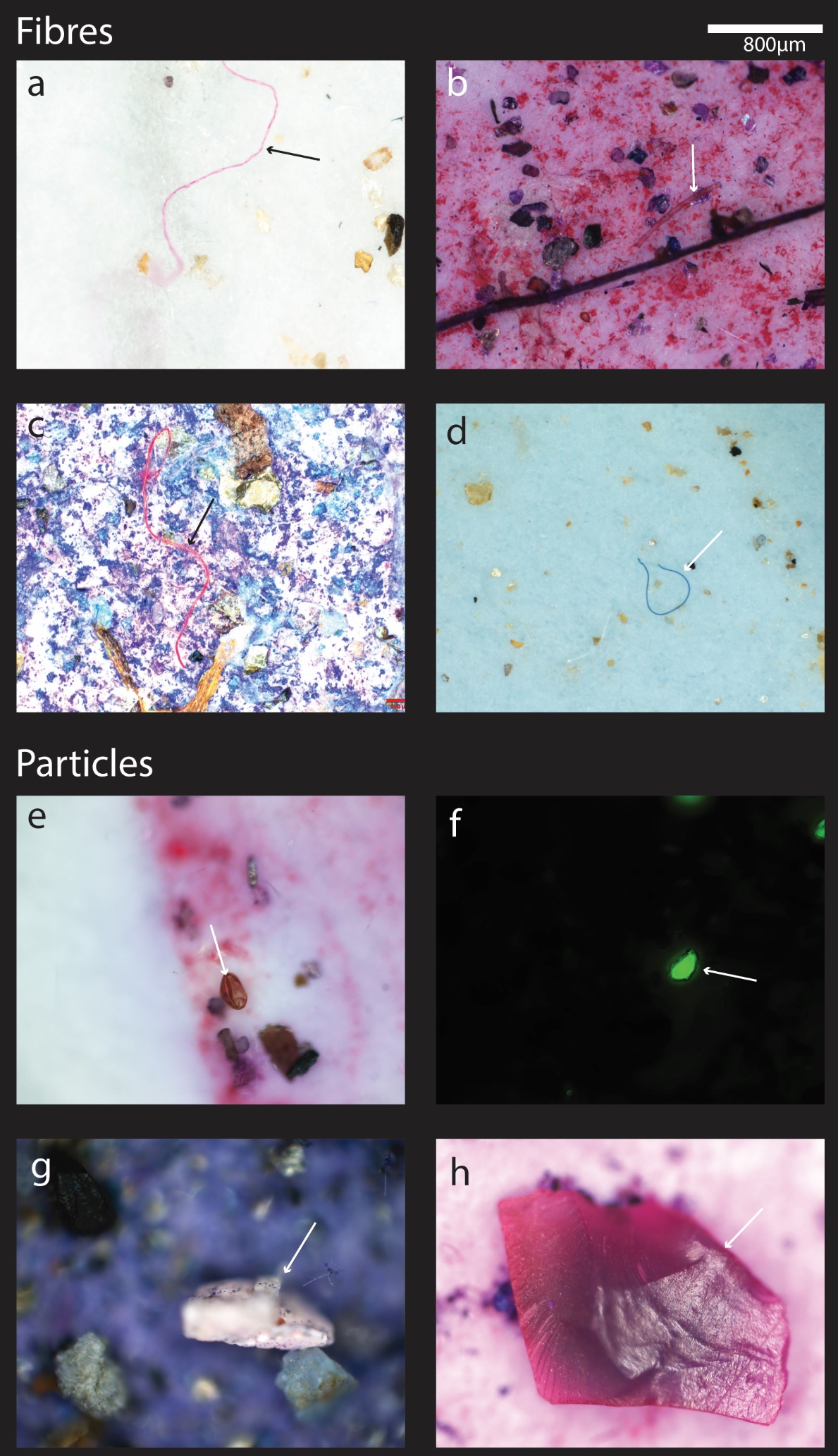


Figure S6: Photographs taken in bright field and fluorescent light modes showing features described in Figures 6-8 above. a) Ribbon-like natural fibre. b) Natural fibre with unequal thickness. c) & d) Fibres positively identified as microplastic. e) Particle identified as being a seed due to its concave structure. f) Particle showing pale “halo” in fluorescent light. g) Particle of calcium carbonate/chitin with dull, crumbly surface texture. h) Particle positively identified as microplastic. All of the particles have been stained in DI-water containing 5µg mL^-1^ Nile Red.
